# Prevalence and outcome of sepsis in respiratory intensive care unit

**DOI:** 10.1186/s43168-022-00135-9

**Published:** 2022-05-18

**Authors:** Ashraf M. Madkour, Ashraf A. ELMaraghy, Mona M. Elsayed

**Affiliations:** 1 Chest Department, Ain Shams University, Cairo, Egypt; 2 Abbassia Chest Hospital, Cairo, Egypt

**Keywords:** Sepsis, SOFA score, Respiratory ICU

## Abstract

**Background:**

Sepsis is a life-threatening organ dysfunction caused by a dysregulated host response to an infection.

**Objective:**

To assess the prevalence and outcome of sepsis in RICU

**Patients and methods:**

The study was conducted upon 403 patients admitted at RICU of the Abbassia Chest Hospital, Cairo, Egypt; 100 of them had sepsis either on admission or acquired in the RICU during the period from May 2019 to November 2019. Severity of illness was assessed by Acute Physiology and Chronic Health Evaluation II (APACHE II score), which was recorded within 24 h from patient admission. Quick sepsis-related organ failure assessment (qSOFA) score was recorded in emergency room, and sepsis-related organ failure assessment (SOFA) score was recorded on ICU admission and on the 3rd and 7th day of ICU stay. Type of infection (community or hospital acquired), infection site, and pathogenic organisms, all were recorded. Assessment was done also regarding mechanical ventilation, length of RICU stay, the presence of comorbidities, survived patients, and dead ones, as regards causes of death and risk factors.

**Results:**

The study included 100 cases with sepsis out of 403 admitted cases in the same duration with frequency 24%. Among sepsis patients, 72% were males and 28%were females, with mean age 51.62 ± 18.62 years. The main diagnosis was pneumonia (62%), and the main comorbidity was diabetes mellitus (23%). There was significant increase in age among non-survivors when compared with survivors. There was significant increase in number of mechanically ventilated patients and a highly significant incidence of complications and need for vasoactive drugs among non-survivors when compared with survivors. There was a highly significant higher APACHE II score on the 1st day of admission among non-survivor patients. The SOFA score was significantly higher on the 1st day of admission and significantly higher on the 3rd and 7th day of admission among non-survivor patients when compared to survived patients.

**Conclusion:**

The current study showed that sepsis affects nearly one quarter of cases admitted at RICU, and it is usually associated with higher mortality rate in those patients.

**Trial registration:**

ClinicalTrials.gov NCT05240157*.* Registered February 15, 2022. Retrospectively registered.

## Introduction

Sepsis is a life-threatening organ dysfunction caused by a dysregulated host response to infection. The statement further defined septic shock as a “subset of sepsis in which profound circulatory failure, cellular and metabolic abnormalities are associated with a greater risk of mortality than with sepsis alone.” The incidence was estimated to be 270 sepsis cases/100,000 persons/year, with a mortality of 26%. In the last three decades, considerable effort has been expended in improving the recognition and management of sepsis [1].

Several studies have provided epidemiological data on sepsis in critically ill patients in the developed countries with increasing incidence and decreasing mortality*.* However, there is limited information about sepsis in Egypt particularly in respiratory intensive care units (RICUs) [2].

We suggested that more studies are needed to be targeted at septic patients in RICUs for fear of bad outcome and mortality, providing a crucially important data to increase awareness of the national impact of sepsis and highlighting the need for continued research about potential preventive and therapeutic interventions.

## Objective

Assessing the prevalence and outcome of sepsis in respiratory intensive care unit, providing a crucially important data to increase awareness of the impact of sepsis and highlighting the need for continued research into potential preventive and therapeutic interventions.

## Patients and methods

This prospective observational cohort study was conducted upon 403 patients admitted at respiratory intensive care unit (RICU) of Abbassia Chest Hospital, Cairo, Egypt; 100 of them had sepsis either on admission or acquired in the RICU, from 1 May 2019 to 3 November 2019.

- ° *Inclusion criteria*
  Patients ≥ 18 years old with sepsis or septic shock (refer to study tools and definitions) either on admission or acquired in RICU and all episodes of sepsis for the same patient were counted
- ° *Exclusion criteria*
  Patients with length of stay (LOS) at RICU less than 24 h, patients readmitted at RICU during the same hospitalization, patients admitted at RICU post cardiac arrest, or patients with malignancies

### Data collection and recording

On admission, the following was done and recorded for all participants:

1. Detailed medical history including history of previous ICU admission or mechanical ventilation, last antibiotic intake, infected intravenous (IV) or central venous (CV) lines, associated comorbidities and reason of ICU admission
2. Full general and local chest clinical examination
3. Need for vasoactive therapy, fluid balance, and need for renal replacement therapy
4. Laboratory investigations include the following:
  ° Complete blood picture (CBC)
  ° Arterial blood gases analysis (ABGs) on a daily basis
  ° Serum sodium (Na) and potassium (K)
  ° Liver and kidney function tests
  ° Serum lactate (repeated when needed to fulfill criteria for diagnosis of septic shock)
5. Radiological investigations
  ° Chest X-ray (CXR)
  ° Computed tomography chest or brain (when appropriate)
6. Microbiological samples culture and sensitivity (when appropriate): sputum, urine, pleural fluid, or from infected IV line according to the suspected site. Type of infection (community or hospital acquired), infection site (lungs, urinary tract, abdomen, surgical wound), and pathogenic organisms (gram positive, gram negative, atypical bacteria and fungi) were recorded
7. Scores include the following:
  - Quick Sequential Organ Failure Assessment (qSOFA) score was recorded at emergency room [3]
  - Sequential Organ Failure Assessment (SOFA) score was recorded upon RICU admission and on the 3rd and 7th day of ICU stay [4].
  - Acute Physiology and Chronic Health Evaluation II (APACHE II) score was recorded within 24 h from patient RICU admission [3, 5].
8. Recording of the following points:
  Number of patients who were on mechanical ventilation, length of RICU stay, presence of comorbidities, survived patients, and dead ones, as regards causes of death and risk factors
  Complications, e.g., acute respiratory distress syndrome (ARDS), acute kidney injury (AKI), and septic shock

#### Measured outcomes

- Primary: Primary frequency of sepsis among admitted cases, mortality due to sepsis
- Secondary: RICU length of stay due to sepsis, complications due to sepsis, mechanical ventilation due to sepsis, and need for vasoactive agents due to sepsis
- Total RICU mortality was also recorded

#### Management

All patients were subjected to the following management protocol regarding the recent *Surviving Sepsis Campaign Bundle Update* [6]:

1. Measuring lactate level with serial measurement if it was more than 2 mmol/L
2. Blood culture prior to antibiotic administration
3. For patients with sepsis, early empiric broad-spectrum antibiotic therapy initiated antimicrobial coverage included either an extended spectrum beta-lactam, a third- or fourth-generation cephalosporin, or a carbapenem. Additional consideration was paid to methicillin-resistant *Staphylococcus aureus* (MRSA) risk factors, and if present, empiric vancomycin administration was advised. Widespread use of combination therapy, the use of multiple antibiotics with different pharmacodynamic profiles, and mechanisms of action reported a synergistic effect with the addition of an aminoglycoside to a beta- lactam. Combination therapy has been shown to improve survival. Patients with a high risk of mortality such as septic shock received a combination therapy with at least two different classes of antibiotics depending on type of organism, source of infection, and choice of antibiotics kept in mind the most organisms isolated from septic patients
4. Early fluid resuscitation using 30 mL/Kg crystalloid fluid was given for cases of hypotension or when lactate level > 4 mmol/L
5. Perfusion assessment using CVP and central venues oxygen saturation
6. Vasopressor use (norepinephrine was given) for persistent hypotension to maintain MAP ≥ 65 mmHg
7. Adjunctive therapy with steroids (200 mg IV hydrocortisone/day) was given in patients with sepsis who remain hemodynamically unstable despite adequate fluid resuscitation and vasopressor therapy
8. Glycemic control was done when patient’s blood glucose level exceeded 180 mg/dL by administrating insulin

### Study tools and definitions

The Sequential Organ Failure Assessment (SOFA) score was used to demonstrate organ dysfunction [4]. Sepsis was defined as having SOFA score of 2 or more plus evidence of infection. Septic shock was defined when persistent hypotension required the use of vasopressors to maintain a MAP ≥ 65 mmHg and a serum lactate > 2 mmol/L that persisted despite adequate fluid resuscitation [4].

Infection was suspected and confirmed from history, examination, laboratory, and radiological and microbiological investigations. ICU-acquired infection was defined as infection identified at least 48 h after ICU admission, while non-ICU-acquired infection was defined as infection presented on admission or within the first 48 h after ICU admission [7].

ARDS was defined according to Berlin definition [8]. Acute kidney injury was defined as an abrupt (within hours) decrease in kidney function based on an acute decrease of glomerular filtration rate, as reflected by an acute rise in serum creatinine levels and/or a decline in urine output over a given time interval [9].

### Statistical analysis

Data were collected, revised, coded, and entered to the *Statistical Package for Social Science (IBM SPSS) version 23*. The quantitative data were presented as mean, standard deviation, frequency (number of cases and percentage), and ranges when their distribution found parametric and median with interquartile range when their distribution found non-parametric. Qualitative variables were presented as number and percentages. Comparison of numerical variables between the study groups was done using the Student *t*-test and paired *t*-test. The comparison of categorical data was done by using the chi-square test. The level of significance was taken at *p*-value ≤ 0.05 as follows: *p* > 0.05, nonsignificant; *p* < 0.05, significant; and *p* < 0.01, highly significant.

## Results

The total number of patients admitted at RICU during the study period was 403. The total number of patients with sepsis were 100 patients, with an incidence rate of 24.8%. Twenty patients were admitted with sepsis (12 of them had septic shock), 80 patients acquired sepsis (16 of them had septic shock) during RICU admission, and 15 patients had more than one episode of sepsis. The demographic characteristics and scores among sepsis patients are shown in Table 1. All patients had sepsis due to respiratory infections, in which 40 patients had hospital-acquired pneumonia, 4 patients had ventilator-acquired pneumonia, 18 patients had community-acquired pneumonia, 11 patients had empyema, 17 patients had pulmonary TB (new cases), 6 patients had multiple pyemic lung abscesses, 4 patients had bronchiectasis, and 26 patients had acute exacerbation of chronic obstructive pulmonary disease. Causative organisms of infection among sepsis patients are shown in Table 2.

**Table 1:** Demographic data and scores of sepsis patients

**Age**	**Range**	19–85
	**Mean ± SD**	51.620 ± 18.620
**Gender**	**Male**	72 (72.00%)
	**Female**	28 (28.00%)
**qSOFA at ER**	**Range**	2–3
	**Mean ± SD**	2.220 ± 0.416
**APACHE II at 1st day**	**Range**	6–39
	**Mean ± SD**	18.990 ± 5.959
**SOFA score at 1st day**	**Range**	3–13
	**Mean ± SD**	5.530 ± 1.925
**SOFA score at 3rd day**	**Range**	0–14
	**Mean ± SD**	5.000 ± 2.689
**SOFA score at 7th day**	**Range**	0–13
	**Mean ± SD**	5.670 ± 3.321
**Special habits**	**All smokers**	54 (54.00%)
	**Nonsmokers**	46 (46.00%)
	**All addict**	25 (25.00%)
**Previous admission at RICU**	**29**	(29.00%)
**Previous mechanical ventilation**	**17**	(17.00%)
**Diagnosis**	**Pneumonia**	62 (62.00%)
	**AECOPD**	26 (26.00%)
	**Pulmonary tuberculosis**	17 (17.00%)
	**Multiple pyemic lung abscesses**	6 (6.00%)
	**Empyema**	11 (11.00%)
	**Bronchiectasis**	4 (4.00%)
**Comorbidity**	**Diabetes mellitus**	23 (23.00%)
	**Hypertension**	11 (11.00%)
	**HCV +ve**	12 (12.00%)
	**Epilepsy**	1 (1.00%)
	**Ischemic heart disease**	19 (19.00%)
	**HIV**	11 (11.00%)
	**Rheumatoid arthritis**	1 (1.00%)
	**Old stroke**	2 (2.00%)
	**Old intracranial hemorrhage**	1 (1.00%)

*SD* standard deviation, *SOFA* Sequential Organ Failure Assessment, *APACHE* Acute Physiology and Chronic Health Evaluation, *RICU* respiratory intensive care unit, *AECOPD* acute exacerbation of chronic obstructive airway disease, *HCV +ve* hepatitis C virus positive, *HIV* human immunodeficiency virus, *ER* emergency room, *qSOFA* Quick Sequential Organ Failure Assessment

**Table 2:** Type of causative organism of infection among sepsis cases

Type of causative organism of infection	Number of patients (%)
***Klebsiella*** **species**	29 (29%)
***Pseudomonas*** **species**	19 (19%)
**Acid fast bacilli**	17 (17%)
***Acinetobacter*** **species**	16 (16%)
***Candida***	14 (14%)
***Staphylococcus aureus*** **species**	10 (10%)
***Escherichia coli*** **species**	8 (8%)
***Proteus*** **species**	2 (2%)

*%* percentage

Measured outcomes of sepsis patients are shown in Table 3. The total RICU mortality was 220 patients (54.9%). Out of 100 patients with sepsis, 68 patients died (68%) representing 16.8% of total number of patients admitted at RICU and 30.9% of total mortality at RICU. There was statistically significant older age among non-survivors from sepsis when compared with survivors, with *p*-value = 0.021. There was no statistically significant difference found between survivors and non-survivors from sepsis as regards type of infection and causative organisms.

**Table 3:** Measured outcomes among sepsis cases

**Length of stay due to sepsis (days)**	**Range:** 7–45 **Mean ± SD:** 12.720 ± 7.55	
**Complications due to sepsis**	**Septic shock**	28%
	**AKI**	8%
	**ARDS**	7%
**Mechanical ventilation due to sepsis**	59%	
**Need of vasoactive drugs due to sepsis**	28%	
**Primary frequency of sepsis among admitted cases**	24.8%	
**Mortality due to sepsis**	68%	

*SD* standard deviation, % Percentage, *AKI* acute kidney injury, *ARDS* acute respiratory distress syndrome

There was statistically significant mortality among mechanically ventilated patients due to sepsis, with *p*-value = 0.010, and a highly statistically significant incidence of complications and need for vasoactive drugs among non-survivors from sepsis when compared with survivors, but there was no statistically significant difference found between survivors and non-survivors as regards length of ICU stay as shown in Table 4. There were statistically significant impaired kidney functions among non-survivors from sepsis when compared with survivors.

**Table 4:** Comparison between survivors and non–survivors from sepsis as regards outcomes

		Outcome				***T***-test or chi-square	
		Survivors		Non-survivors		***T*** or ***X***^**2**^	***p***-value
**Length of ICU stay (days)** **Mean ±SD**		11.188 ± 5.152		13.441 ± 8.389		−1.399	0.165
**Mechanically ventilated**		13	40.63%	46	67.65%	6.568	0.010*
**Complications**	**Septic shock**	0	0.00%	28	41.18%	18.301	< 0.001*
	**AKI**	0	0.00%	8	11.76%	4.092	0.043*
	**ARDS**	0	0.00%	7	10.29%	3.542	0.060
**Need for vasoactive**	**Yes**	0	0.00%	28	41.18	18.301	< 0.001*

*RICU* respiratory intensive care unit, *MV* mechanical ventilation, *AKI* acute kidney injury, *ARDS* adult respiratory distress syndrome

* Chi-square

There was a highly statistically significant higher APACHE II score on the 1st day of admission among non-survivors from sepsis, with *p*-value < 0.001 as shown in Table 5.

**Table 5:** Comparison between survivors and non-survivors from sepsis as regards APACHE II score on the 1st day of admission

		Outcome		***T***-test	
		Survivors	Non-survivors	***t***	***p***-value
**APACHE II score on 1st day**	**Range**	**6 − 26**	**7 − 39**	**−3.737**	**< 001**
	**Mean ± SD**	**15.938 ± 4.996**	**20.426 ± 5.862**		

*APACHE II* Acute Physiology and Chronic Health Evaluation II

There was no statistically significant difference found between survivors and non-survivors from sepsis as regards quick SOFA score at emergency room. The SOFA score was significantly higher on the 1st day of admission and highly significantly higher on the 3rd and 7th day of admission among non-survivors when compared to survivors, with *p*-value < 0.001. In addition, there was highly statistically significant increase in the score in the non-survivors from sepsis on comparing the score on the 1st day of admission with that on the 3rd and 7th day and highly statistically significant reduction of the score in the survivors on comparing the score on the 1st day of admission with that on the 3rd and 7th day, with *p*-value < 0.001 as shown in Table 6.

**Table 6:** Comparison between survivors and non-survivors from sepsis as regards SOFA score on admission, 3rd and 7th day of admission

SOFA		Outcome		***T***-test	
		Survivors	Non-survivors	***t***	***p***-value
At 1st day	Range	3 **−** 10	3 **−** 13	**−**2.150	0.034
	Mean ± SD	4.938 ± 1.917	5.809 ± 1.879		
At 3rd days	Range	0 **−** 8	2 **−** 14	**−**4.432	< 0.001
	Mean ± SD	3.406 ± 1.982	5.750 ± 2.662		
At 7th days	Range	0 **−** 6	2 **−** 13	**−**8.502	< 0.001
	Mean ± SD	2.531 ± 1.367	7.147 ± 2.918		
1–3 D	Differences	1.531 ± 1.796	0.059 ± 2.461		
	Paired test	< 0.001*	0.844		
1–7 D	Differences	2.406 ± 1.998	**−**1.338 ± 2.915		
	Paired test	< 0.001*	< 0.001*		
3–7 D	Differences	0.875 ± 1.737	**−**1.397 ± 2.604		
	Paired test	0.008*	< 0.001*		

*SOFA* Sequential Organ Failure Assessment

## Discussion

Severe sepsis and septic shock carry high potential mortality rates, possibly up to 40–50% [10]. *The Global Burden of Disease Study* reported in 2017 an estimate that 48.9 million incident cases of sepsis were recorded worldwide, and 11 million sepsis-related deaths were reported, representing 19.7% of all global deaths [11]. According to these data, the prevalence of sepsis in this study was 24.8%, which came partially in agreement with the report of the *Worldwide Data from the Intensive Care Over Nations Audit* published in 2018, which included 10,069 patients, from Europe (54.1%), Asia (19.2%), America (17.1%), and other continents (9.6%). Sepsis was identified during the ICU stay in 2973 (29.5%) patients [12]. On the other hand, the prevalence in our study did not match with Heldens et al. (2018) [13] who stated that the incidence of sepsis and septic shock was 16.9% and 5.7%, respectively, among 864 patients admitted at the ICU over 3 months’ duration. In addition, Valentine and his colleagues [14] reported that the incidence of sepsis and septic shock in ICUs was estimated to be 101.8 and 19.3 per 100,000 patients/year, respectively. This mismatch might be due to the difference in number and cause of admission to ICU of enrolled patients in each study, putting in mind that our patients had significant burden of respiratory infections.

In the current study, 72% of the cases with sepsis were males, and 28% were females, with mean age 51.620 ± 18.620 years; the mean age of cases who survived from sepsis was 45.406 ± 17.996 years that was statistically significant lower when compared with non-survivors; as the mean age was 54.544 ± 18.312 years, most of cases within two groups were males (71.88% in the survivors and 72.06% in the non-survivors), but sex distribution did not reveal significant difference. This issue resembled the study conducted by Ortiz et al. [15] who found that the average age of the septic patients was 54.5 ± 20 years, which indicated that sepsis was more common in older age. However, in Ortiz et al. [15] study, males represented 53% of cases, and females represented 47%. The difference in results might be because their study included all Columbian intensive cares, i.e., larger population. This partially came in agreement with the following studies: Martin et al., van Gestel et al., and Finfer et al. [16–18] who stated that the mean age of patients with severe sepsis (with fatal outcomes) in most epidemiological studies ranged between 55 and 64 years.

Angus et al. [19] found direct relationship between advanced age and the incidence of severe sepsis and septic shock, with a marked increase in incidence in elderly individuals. In general, incidence of sepsis had clearly increased, probably due to progressive aging of population, provided that several studies had demonstrated a relationship between age and incidence of sepsis and a larger number of people with disease comorbidities.

Among the cases with sepsis in our study, 20% were admitted already with sepsis, and 80% of patients acquired sepsis at our RICU. This matched partially with Baharoon et al. [20] study, where 60% of cases were hospital-acquired infections, while 40% were community acquired. Respiratory infections are known to be the most common cause of ICU admission in almost all the healthcare facilities all over the world [21]. This agreed with the study conducted by Baykara et al. [22] who stated that the most common site of infection was the respiratory system (71.6%). Approximately, 32.8% of all infected patients had community-acquired infections, whereas 54.4% had nosocomial infections.

In our study, the mean length of RICU stay was 12.720 ± 7.553 days. The percentage of cases who needed mechanical ventilation was 59% among the septic patients. Mechanical ventilation had a statistical significance with mortality, where mechanical ventilation was more frequent in non-survivors than in survivors. In the study of Fialkow et al. [23], out of 2430 patients admitted at the ICU, 46% of patients needed mechanical ventilation with a mortality rate 51%.

In the present study, gram-negative bacteria were the most common causative organism of infection, followed by acid fast bacilli, fungal infection, and gram-positive bacteria. The most common isolated organism was *Klebsiella* followed by *Pseudomonas*. This agreed with the recent results of the Egypt’s hospital-acquired infection (HAI) surveillance system, where a total of 3836 Enterobacteriaceae isolates from 3109 ICU patients were included in the surveillance system from 2011 to 2017. Isolates were collected from blood, urine, wound, or respiratory specimen on or after day 3 of ICU admission. *Klebsiella* were the most isolated pathogen (*n* = 929), and it was reported to be responsible for resistance for carbapenem [24]. This was also supported with the report of the *Worldwide Data from the Intensive Care over Nations Audit* published in 2018, which showed that according to the results of culture and sensitivity analysis in the patients who developed ICU sepsis, methicillin-resistant *Staphylococcus aureus* (MRSA) was more common in the Middle East (14.4%) and North America (12.8%) than in Western Europe (6.1%). *Klebsiella* isolates were most commonly reported in Africa (31.3%), Eastern Europe (28.5%), and South America (24.7%), and *Pseudomonas* was most frequent in Eastern Europe (21.1%) and South America (20.4%). Fungal organisms contributed to 14.5% and 14.8% of isolates in Western and Eastern Europe, respectively, but to only 5.1% of isolates in North America [12].

The mortality rate in our study was 68% among the septic patients admitted at RICU, and the overall mortality rate among all the cases admitted at RICU was 54.9%. In agreement with our results, the hospital mortality rate of sepsis was also found to be high (44.5%) in Asia according to a study performed at 150 ICUs from 16 countries in 2011 [25]. Similar results were also reported by a study conducted by Melville et al. [26], and from the beginning of 2005 to the end of October 2014, mortality rate in septic patients was (44.6%). This high mortality from sepsis in ICU might be due to increased incidence of sepsis and its complications that led to increased mortality rate. Sakr et al. and Abe et al. [12, 27] showed that the overall ICU mortality rate was 23.4% in a large study included 1184 adults recruited from 59 ICUs. They reported that for pneumonia, mortality rate was 28.8% in all cases, 33.9% in cases with septic shock, and 19.6% in cases without septic shock. Shankar-Hari Manu et al. [28] published a systematic review that identified a crude mortality rate associated with septic shock of 47%. The mortality rate of sepsis might be different among countries and continents due to differences in the provision of intensive care facilities and treatments, as well as the number of included patients in each report.

In our study, DM was the most common associated chronic disease that was found in 18.75% of survivors and in 25% of non-survivors, followed by ischemic heart disease (IHD) that was found in 12.5% of survivors and in 19.2% of non-survivors. Smoking was the most common risk factor that was found in 50% of non-survivors and in 62.5% in survivors, with no statistically significant difference in between. This came in accordance with Jandial et al. [29] who showed that DM was the most common associated chronic disease in their study that was found in 31.4% of septic patients who survived and in 38.6% of the non-survivor sepsis group. The authors also reported that smoking was the most common risk factor among the cases included in their study (32.6% vs 39.5% in the two groups, respectively). In our study, HTN was present in 6.25% of survivors and in 13.25% of non-survivors; however, Kim et al. [30] showed that HTN was more prevalent than DM in the cases included in their study (64.6% of the cases in the survivor group and 52.3% of the cases in the non-survivor group). Similar results were revealed by Shaikh and Yadavalli [10] who showed that DM (39.5%) and HTN (34.5%) were most common comorbid conditions among the cases included in their study.

In the current study, the tested laboratory parameters did not reveal a statistically significant difference between the survivors and non-survivors, except the serum urea and serum creatinine, which was statistically significantly higher in non-survivors (*p* = 0.016 and 0.012, respectively).

In the present study, there was a highly statistically significant higher APACHE II score on the 1st day of admission among non-survivor septic patients when compared with the survivors. Similar results were reported by Pandya et al. [31] as they revealed that APACHE II score was significantly higher in those who died.

In this study, the SOFA score was significantly higher on the 1st day of admission and highly significantly higher on 3rd and 7th day of admission among non-survivors when compared to survivors. Also, there was highly statistically significant increase in the score in the non-survivors on comparing the score on the 1st day of admission with that on the 3rd and 7th day and highly statistically significant reduction of the score in the survivors on comparing the score on the 1st day of admission with that on the 7th day. This came in agreement with Jain et al. [32] study, where SOFA score on the 1st day of admission, on 3rd day, and on 7th day was statistically significantly higher in non-survivors than in survivors.

Limitations of the current study include the small sample size and the fact that it was a single-center study, which might decrease the power of the results, in addition to unavailability of cultures for anerobic organisms.

Our study is among the very few studies from Egypt that addressed the occurrence and outcome of sepsis in respiratory intensive care units. It showed that sepsis affects nearly one quarter of cases admitted at RICU, and it is usually associated with higher mortality rate in those patients, especially in older age, and with mechanical ventilation and mortality was associated with higher incidence of complications and septic shock.

## Data Availability

Tables included.

## Abbreviations

RICU: Respiratory intensive care unit
LOS: Length of stay
IV: Intravenous
CV: Central venous
CBC: Complete blood count
ABGs: Arterial blood gases
Na: Sodium
K: Potassium
CXR: Chest X-ray
qSOFA: Quick Sequential Organ Failure Assessment
SOFA: Sequential Organ Failure Assessment
APACHE II: Acute Physiology and Chronic Health Evaluation II
ARDS: Acute respiratory distress syndrome
AKI: Acute kidney injury
MRSA: Methicillin-resistant *Staphylococcus aureus*
MAP: Mean arterial pressure
ICU: Intensive care unit
PaO_2_/FiO_2_ ratio: Ratio of arterial oxygen partial pressure (PaO2 in mmHg) to fractional of inspired oxygen (FiO_2_ expressed as a fraction, not a percentage)
PEEP: Positive end-expiratory pressure
CPAP: Continuous positive airway pressure
HAI: Hospital-acquired infection
DM: Diabetes mellitus
IHD: Ischemic heart disease
HTN: Hypertension
IRB: Institutional Review Board
HIV: Human immunodeficiency virus
ER: Emergency room
SD: Standard deviation
MV: Mechanical ventilation
